# YOLO-DCRCF: An Algorithm for Detecting the Wearing of Safety Helmets and Gloves in Power Grid Operation Environments

**DOI:** 10.3390/jimaging11090320

**Published:** 2025-09-19

**Authors:** Jinwei Zhao, Zhi Yang, Baogang Li, Yubo Zhao

**Affiliations:** 1 Department of Electronic and Communication Engineering, North China Electric Power University, Baoding 071003, China; yangzhi@ncepu.edu.cn (Z.Y.); baogangli@ncepu.edu.cn (B.L.); 220232215067@ncepu.edu.cn (Y.Z.); 2 Hebei Key Laboratory of Power Internet of Things Technology, North China Electric Power University, Baoding 071003, China; 3 Hebei Engineering Research Center of Intelligent Technology for Power Internet of Things, North China Electric Power University, Baoding 071003, China

**Keywords:** YOLOv11, safety helmet and glove wearing detection, deformable convolutional network, recalibration feature pyramid network

## Abstract

Safety helmets and gloves are indispensable personal protective equipment in power grid operation environments. Traditional detection methods for safety helmets and gloves suffer from reduced accuracy due to factors such as dense personnel presence, varying lighting conditions, occlusions, and diverse postures. To enhance the detection performance of safety helmets and gloves in power grid operation environments, this paper proposes a novel algorithm, YOLO-DCRCF, based on YOLO11 for detecting the wearing of safety helmets and gloves in such settings. By integrating Deformable Convolutional Network version 2 (DCNv2), the algorithm enhances the network’s capability to model geometric transformations. Additionally, a recalibration feature pyramid (RCF) network is innovatively designed to strengthen the interaction between shallow and deep features, enabling the network to capture multi-scale information of the target. Experimental results show that the proposed YOLO-DCRCF model achieved mAP50 scores of 92.7% on the Safety Helmet Wearing Dataset (SHWD) and 79.6% on the Safety Helmet and Gloves Wearing Dataset (SHAGWD), surpassing the baseline YOLOv11 model by 1.1% and 2.7%, respectively. These results meet the real-time safety monitoring requirements of power grid operation sites.

## 1. Introduction

In the complex and high-risk environment of power grid operations, safety helmets and gloves serve as critical protective equipment, significantly reducing the likelihood of worker injuries. Due to the intricate and variable nature of the power grid work environment—including dense personnel presence, varying lighting conditions, occlusion issues, posture changes, diverse background settings, dynamic operations, and the variety of protective equipment—traditional detection methods for safety helmets and gloves exhibit low accuracy. Consequently, detection methods capable of accurately identifying and localizing safety helmets and gloves under adverse conditions require greater robustness and adaptability. This, in turn, contributes to creating a safer work environment, ultimately reducing the occurrence of occupational injuries.

Traditional object detection algorithms are broadly classified into two-stage and one-stage approaches. Two-stage methods, exemplified by R-CNN [1], Faster R-CNN, and R-FCN [2], first generate a set of candidate regions potentially containing target objects in the input image using techniques such as selective search. Subsequently, a pretrained convolutional neural network (e.g., AlexNet or VGG16) extracts features from each candidate region. These features are then used for classification and bounding box regression. However, two-stage algorithms incur significant computational overhead due to the need for individual feature extraction and classification for each candidate region. Additionally, their training process is complex, requiring separate models for the convolutional neural network, support vector machine (SVM), and bounding box regressor.

One-stage object detection algorithms, primarily represented by SSD [3] and YOLO [4], employ a single neural network to directly predict the locations and categories of objects from input images. This approach eliminates the complex process of generating candidate regions [5], thereby achieving faster detection speeds [6]. To develop a digital safety helmet monitoring system, Zhou et al. [7] proposed a safety helmet detection method based on YOLOv5. Similarly, Li et al. [8] introduced an SSD-MobileNet algorithm, utilizing a convolutional neural network for real-time safety helmet detection at construction sites. Fan et al. [9] proposed a feature-enhanced lightweight algorithm, LG-YOLOv8, for safety helmet detection. Additionally, Lin et al. [10] developed a mosaic data augmentation technique to improve the detection accuracy of small objects.

Considering the specific task of detecting the wearing of safety helmets and gloves, as well as the challenges posed by varying object sizes and limited computational resources in real-world power grid operation environments, this study proposes a novel algorithm, YOLO-DCRCF, for detecting the wearing of safety helmets and gloves in such settings. The algorithm aims to accurately identify whether power grid personnel are properly wearing safety helmets and gloves. The primary contributions of this study are summarized in the following three points:

- By introducing the second version of the deformable convolutional network (DCNv2), additional deformable transformation layers are stacked to further enhance the geometric transformation modeling capabilities of the entire YOLOv11 network, thereby better adapting to variations in the scale, posture, and viewpoint of the detection objects.
- This paper designs a recalibrated feature pyramid (RCF) network that promotes the transfer of information between features by introducing a bidirectional fusion mechanism between high- and low-resolution features, further improving the effect of multi-scale feature fusion. Combined with an adaptive attention mechanism, the RCF network dynamically adjusts feature weights based on feature maps of different resolutions and content, finely delineating object contours and recalibrating object positions through the selective aggregation of boundary and semantic information, accurately capturing the multi-scale features of the target.
- A specialized dataset for safety helmet and glove wearing detection (SHAGWD) is constructed, and the effectiveness and reliability of the YOLO-DCRCF model are verified on data from diverse scenes. Experimental results indicate that the model can stably perform safety helmet and glove wearing detection in power grid operation environments, providing a new technical solution for the field of power grid operation safety monitoring.

The remainder of this paper is organized as follows. Section 2 introduces related research work. Section 3 describes the datasets used for the experiments. Section 4 presents the safety helmet and glove wearing detection algorithm for power grid operation environments. Section 5 describes the experimental process and analysis of the experimental results to evaluate the performance of the proposed algorithm. Finally, Section 6 concludes the paper and offers prospects for future work.

## 2. Related Work

In power grid operation environments, the use of safety helmets and gloves is essential for ensuring personnel safety. A safety helmet detection method based on Faster R-CNN, which treats individuals wearing safety helmets as positive samples, enhances robustness across various postures [11]. Enhancements to the YOLOv5 algorithm, including a lightweight version and a multi-category training strategy that classifies individuals with complete safety equipment as positive samples and those lacking proper personal protective equipment as negative samples, significantly reduce the false positive rate [12]. Incorporating Reverse Progressive Attention (RPA) into the SSD framework improves detection performance for small targets [13]. The integration of Adaptive Spatial Feature Fusion (ASFF) and Deformable Convolutional Network version 2 (DCNv2) into the detection head enables the network to more effectively capture multi-scale target information, addressing challenges posed by the small size of helmets in images [14]. To mitigate the impact of occlusions on helmet detection accuracy, the C-ELAN module employs deformable convolutions to extend the receptive field, thereby providing rich contextual feature information for coordinate attention and improving the network’s ability to accurately recognize target locations [15]. For practical deployment, the YOLO-ESC model enables real-time monitoring of workers’ helmet usage via drones and other platforms while automatically streamlining the detection results from video streams [16].

The method that combines YOLO with Transformer possesses stronger data fitting capabilities, generalization, and scalability [17]. The C3TR module based on a self-attention mechanism can establish direct connections between any two points in an image, thus better understanding the content of the image [18]. CAST-YOLO utilizes cross-attention to eliminate the impact of domain-invariant feature shifts on cross-domain target detection, thereby improving the accuracy of foggy day adaptive detection [19]. To address issues such as high resolutions, poor imaging clarity, and significant size differences between targets, the YOLO11 algorithm introduces the MSDA module to construct the dependency relationship between sparse features and distant pixels, and it combines the CSPStage module to enhance the capture of multi-scale semantic information [20].

## 3. Dataset

This paper employs the Safety Helmet Wearing public dataset SHWD for model training, validation, and research experimental testing. This dataset is renowned for its use in safety helmet and human head detection, encompassing a variety of real-world scenarios of wearing safety helmets in complex work environments. It includes 9044 instances of human safety helmet wearing (positive samples) and 111,514 normal head instances (negative samples). The dataset used in the experiments was divided into a training set (5306 images), a validation set (1593 images), and a test set (682 images).

**Figure 1:**
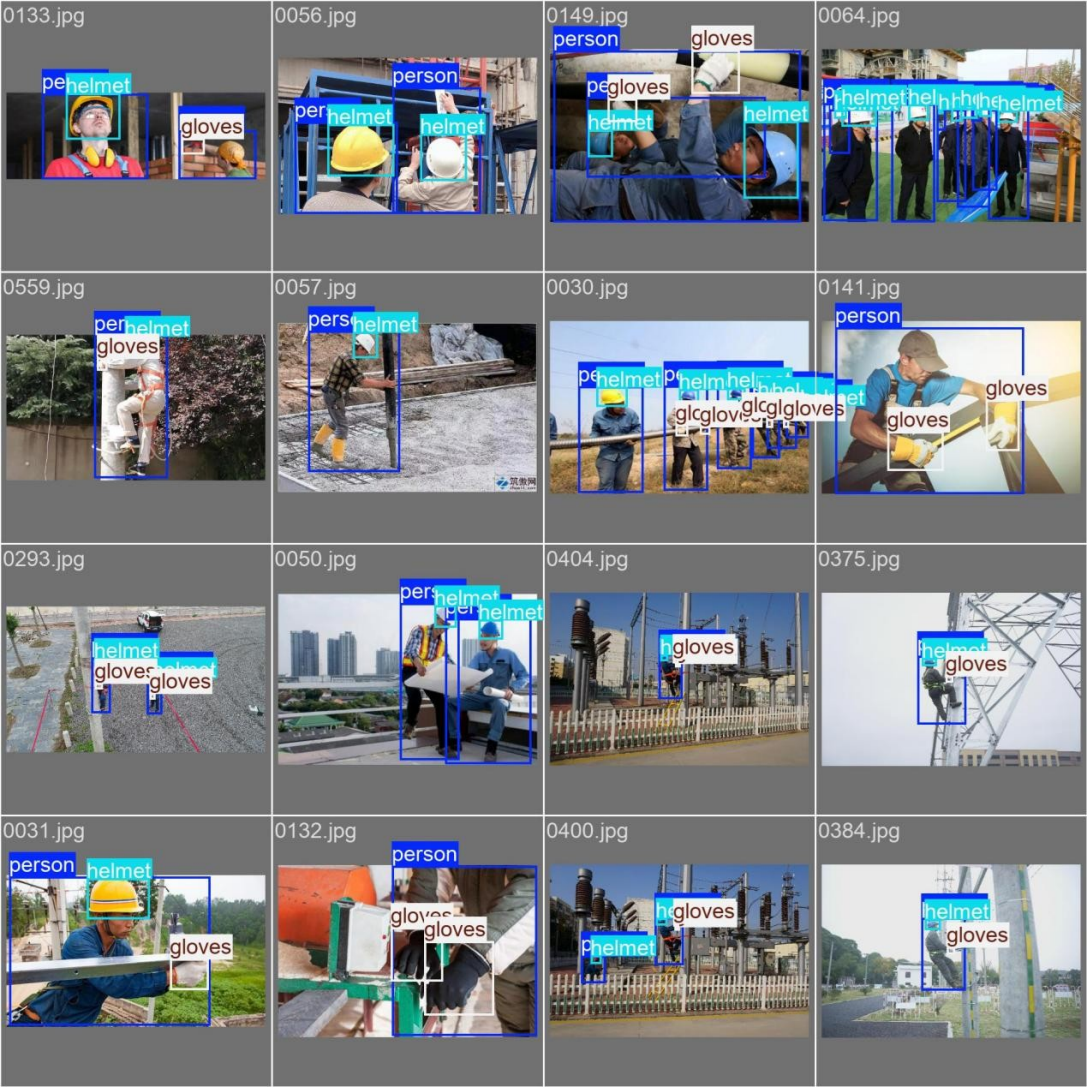
These are examples of image titles from the SHAGWD dataset.

Given the frequent hand movements of personnel during construction, it is crucial to monitor whether they are wearing safety helmets and gloves properly for safety detection during work. Therefore, this paper constructs the Safety Helmet and Gloves Wearing Dataset (SHAGWD), which consists of three categories: safety helmet, gloves, and humans. The dataset contains 934 labels for safety helmets, 675 labels for gloves, and 977 labels for humans. The dataset used in the experiments was divided into a training set (400 images), a validation set (113 images), and a test set (113 images), as shown in Figure 1.

## 4. Algorithm Principles and Improvements

Based on YOLO11, this study introduces a novel algorithm, YOLO-DCRCF, for detecting the wearing of safety helmets and gloves in power grid operation environments, thereby further improving detection performance in these contexts.

### 4.1. YOLO-DCRCF

**Figure 2:**
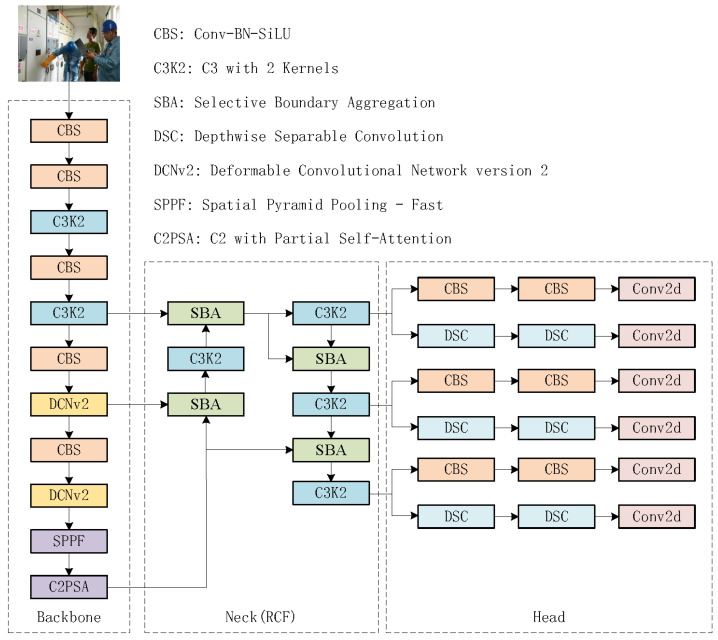
Figure of the overall structure of YOLO-DCRCF.

In this section, the structure of the YOLO-DCRCF algorithm will be introduced. Building upon YOLOv11, the algorithm incorporates the second version of the deformable convolutional network (DCNv2) [21] and the RCF network architecture [22], thereby forming a novel detection algorithm for safety helmets and glove wearing. The overall framework of the algorithm is depicted in Figure 2.

As depicted in Figure 2, the architecture of the YOLO-DCRCF model comprises four components: the input layer, backbone network, neck, and detection head. The input layer resizes input images to meet the training requirements through operations such as scaling, hue adjustment, and mosaic data augmentation, thereby enhancing the diversity of the training data; the backbone network is designed to improve feature extraction by balancing computational efficiency and feature representation, thereby improving the overall performance of the model; the neck facilitates feature fusion by integrating multi-scale feature information via a recalibration feature pyramid network, improving the model’s ability to detect objects of varying sizes and positions; and the detection head generates confidence scores and location information for different targets, utilizing a decoupled head structure to separate detection and classification tasks, thereby not only reducing the model’s parameter count and complexity but also enhancing its generalization and robustness.

### 4.2. Deformable Convolutional Network Version 2 (DCNv2)

**Figure 3:**
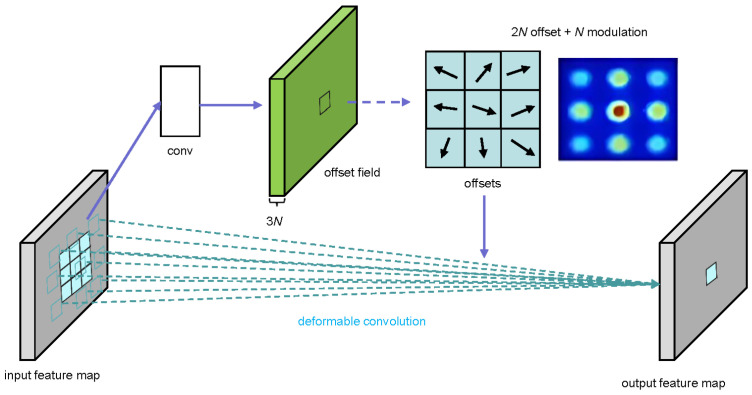
Schematic diagram of the DCNv2 architecture (3 × 3 deformable convolution).

Convolutional neural networks have demonstrated significant success in visual recognition tasks, yet their capacity to model geometric transformations remains limited, primarily depending on data augmentation and model capacity. Deformable convolution improves spatial sampling in convolution and RoI pooling by incorporating additional offsets, as exemplified by DCNv2, shown in Figure 3.

DCNv2 addresses the shortcomings of DCNv1 [23] in two primary aspects. First, it introduces additional convolutional layers capable of learning offsets, allowing the network to manage sampling across a broader range of feature levels. Second, it introduces a modulation mechanism that enables each sample to be subject to both learned offsets and modulation by learned feature amplitudes, thus enhancing the network’s capacity to manipulate spatial support regions.

For the modulated deformable module, given a convolution kernel with *K* sampling locations, let $w_{k}$ and $p_{k}$ denote the weight and the pre-specified offset for the *k*th location, respectively. Let x(p) and y(p) represent the feature at position p in the input feature map x and the output feature map y, respectively. The modulated deformable convolution can be expressed as shown in Equation (1):

(1) $$y\left(p\right)=\sum_{k=1}^{K}w_{k}\times x\left(p+p_{k}+\Delta p_{k}\right)\times \Delta m_{k}$$

In Equation (1), $\Delta p_{k}$ and $\Delta m_{k}$ represent the learnable offset and modulation scalar at the *k*th location, respectively. The value of $\Delta m_{k}$ ranges from 0 to 1, while $\Delta p_{k}$ is an unconstrained real number. Both variables are obtained through separate convolutional layers applied to the same input feature map *x*.

The design of modulated deformable RoI pooling is similar. Given an input RoI, RoI pooling divides it into K spatial bins. Within each bin, a sampling grid with uniform spatial intervals is applied, and the average of the sampled values on the grid is computed. The computation method is shown in Equation (2):

(2) $$y\left(k\right)=\sum_{j=1}^{n_{k}}x\left(p_{kj}+\Delta p_{k}\right)\times \frac{\Delta m_{k}}{n_{k}}$$

In Equation (2), $p_{kj}$ denotes the sampling position of the jth grid cell within the kth bin, and $n_{k}$ represents the number of sampling grid cells. The value obtained from the feature $x\left(p_{kj}+\Delta p_{k}\right)$ is used for bilinear interpolation, and this value is generated by the sibling branch on the input feature map.

### 4.3. RCF (Recalibrated Feature) Network

**Figure 4:**
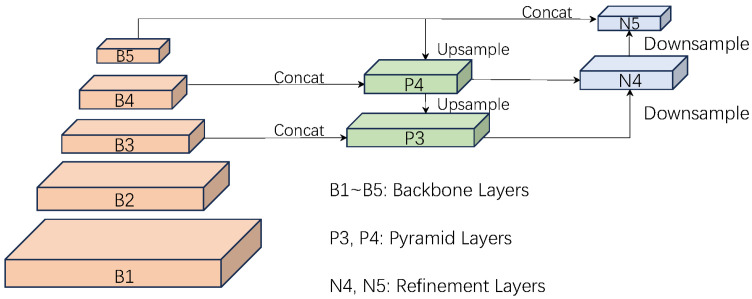
Feature pyramid network architecture.

In the field of deep learning for computer vision, the feature pyramid network (FPN) is a widely adopted method for multi-scale feature fusion, with its architecture depicted in Figure 4. However, the traditional FPN structure has inherent limitations, including insufficient semantic information in shallow features, which causes detail loss in deep features, leading to localization errors and false negatives due to intra-layer spatial information loss and semantic disparities between layers [24]. High-level semantic information in the FPN becomes diluted during downward propagation, and the diverse semantic requirements of different scale layers limit lower layers to accessing only minimal semantic information. Concurrently, the absence of spatial information in high-level features hinders accurate bounding box localization. The dilution of low-level semantic information combined with the deficiency of high-level spatial information results in an information imbalance across the feature pyramid, thereby diminishing the network’s overall performance.

To address these challenges, the present study introduces a novel recalibrated feature pyramid network (RCF network) that integrates a Selective Boundary Aggregation (SBA) module to selectively aggregate boundary and semantic information, thereby enhancing feature interactions [25]. The SBA module facilitates information transmission via bidirectional fusion of high- and low-resolution features, leveraging the semantic information of deep features to enhance the capability of shallow features in recognizing complex objects while utilizing the detailed information of shallow features to improve object boundary delineation and the resolution of deep features. The bidirectional fusion mechanism ensures comprehensive information transfer between features, thereby improving the effectiveness of multi-scale feature fusion. Furthermore, the SBA module dynamically adjusts feature weights based on the diverse resolutions and contents of feature maps, thereby enabling more effective capture of the target’s multi-scale features.

**Figure 5:**
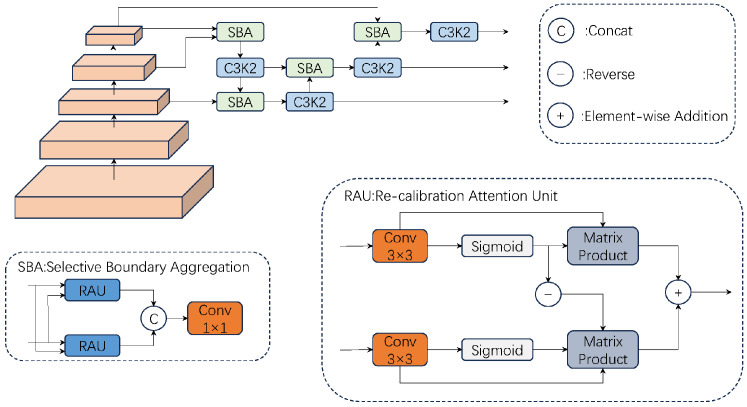
The RCF network’s structure.

The RCF network structure, incorporating the SBA module, effectively mitigates the shortcomings of a traditional FPN in feature fusion, thereby enhancing the interaction between high- and low-resolution features. In comparison with the traditional FPN structure, the SBA module more effectively captured the target’s multi-scale features, thus enhancing the model’s overall performance. The architecture of the RCF network is depicted in Figure 5.

## 5. Experimental Section

### 5.1. Experimental Details

The experimental set-up of this paper is shown in Table 1.

For the model training, the following parameters were configured:

- Input image size: 640 × 640;
- Training epochs: 300 complete iterations over the training dataset;
- Batch size: 32 images per batch;
- Data loading threads: 8 workers used to accelerate data reading and preprocessing;
- Optimizer: stochastic gradient descent (SGD).

All experiments were conducted without using pretrained weights to ensure the purity of the results.

### 5.2. Experiment and Analysis on the Public Safety Helmet Wearing Dataset (SHWD)

To validate the performance of the safety helmet and glove wearing detection algorithm YOLO-DCRCF proposed in this paper for the power grid working environment, a comparative experiment was conducted on the Public Safety Helmet Wearing Dataset (SHWD), using YOLO11 as the baseline model. The evaluation metrics for the model included precision, recall, mAP50, and mAP50-95:

(3) $$\mathrm{Precision}=\frac{TP}{TP+FP}$$

(4) $$\mathrm{Recall}=\frac{TP}{TP+FN}$$

The average precision (AP) for a single class is defined as the average of all precision values across all possible recall values:

(5) $$\mathrm{AP}=\int_{0}^{1}P\left(r\right)\;dr$$

(6) $$\mathrm{mAP}=\frac{1}{m}\sum_{i=1}^{m}AP_{i}$$

Meanwhile, mAP50 denotes the mean average precision (AP) across all object classes at an IoU threshold of 0.5. The “m” explicitly represents the arithmetic average of the AP values over all categories. For example, in the SHWD, we evaluated two classes: “helmet” and “person”. In the SHAGWD, the evaluation included three classes: “person”, “helmet”, and “gloves”. Here, mAP50-95 is a weighted average of the AP values over the IoU threshold range from 0.5 to 0.95, providing a more comprehensive and stringent evaluation. To rigorously evaluate the generalization performance of the proposed algorithm, a 5-fold cross-validation strategy was adopted in this study. The original training set was randomly partitioned into five mutually exclusive subsets. In each training round, four subsets were used for model training, while the one remaining subset was reserved for validation. The final performance metrics are reported as the mean ± standard deviation across the five validation rounds, computed according to the following formula:

(7) $$\bar{\mathrm{mAP}}=\frac{1}{5}\sum_{k=1}^{5}{\mathrm{mAP}}^{\left(k\right)}$$

(8) $$\sigma_{\mathrm{mAP}}=\sqrt{\frac{1}{5}\sum_{k=1}^{5}{\left({\mathrm{mAP}}^{\left(k\right)}-\bar{\mathrm{mAP}}\right)}^{2}}$$

**Figure 6:**
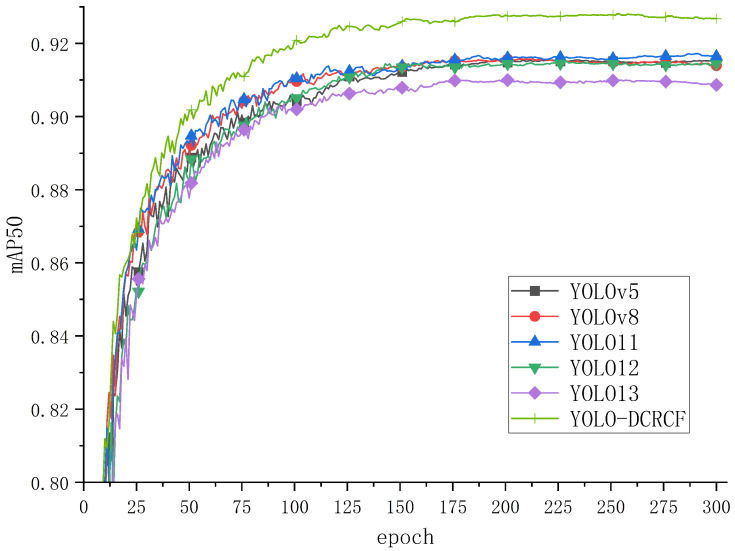
The model was trained on the SHWD, achieving the shown mAP50 values.

This method effectively mitigated evaluation bias caused by the randomness of data partitioning and ensured the statistical reliability of the experimental results. The mAP50 curves during the training process for various models are illustrated in Figure 6:

Figure 6 depicts the training curve of the model on the public Safety Helmet Wearing Dataset (SHWD), where the horizontal axis represents the number of iterations and the vertical axis denotes the mAP50. Analysis of Figure 6 indicates that YOLO11 achieved the highest validation mAP50 among the baseline models, demonstrating its superior suitability as a foundational model for safety helmet wearing detection. The YOLO-DCRCF model, proposed in the present study, significantly surpassed other baseline models in terms of mAP50 after training for 30 epochs. The experimental results effectively confirm that the integration of DCNv2 significantly enhanced the geometric transformation modeling capabilities of the YOLO11 network. Moreover, the bidirectional fusion mechanism between high- and low-resolution features in the RCF network, coupled with an adaptive attention mechanism, facilitated more effective capture of the target’s multi-scale features, resulting in enhanced accuracy in safety helmet identification and underscoring the superiority of the proposed model in the safety helmet wearing recognition task.

Table 2 presents the test results of various models on the public Safety Helmet Wearing Dataset (SHWD), with the following specific analyses:

1. In comparison with the baseline YOLO11 model, the YOLO-DCRCF model, proposed in the present study, demonstrated improvements of 0.7% in precision, 0.9% in recall, 1.1% in mAP50, and 2.1% in mAP50-95.
2. Although the YOLO-DCRCF model exhibited lower precision than YOLO13, it surpassed YOLO13 in all other metrics, demonstrating that the proposed algorithm is better suited for applications such as safety monitoring, thus confirming its superior performance.

### 5.3. Safety Helmet and Gloves Wearing Dataset (SHAGWD) Experiment and Analysis

**Figure 7:**
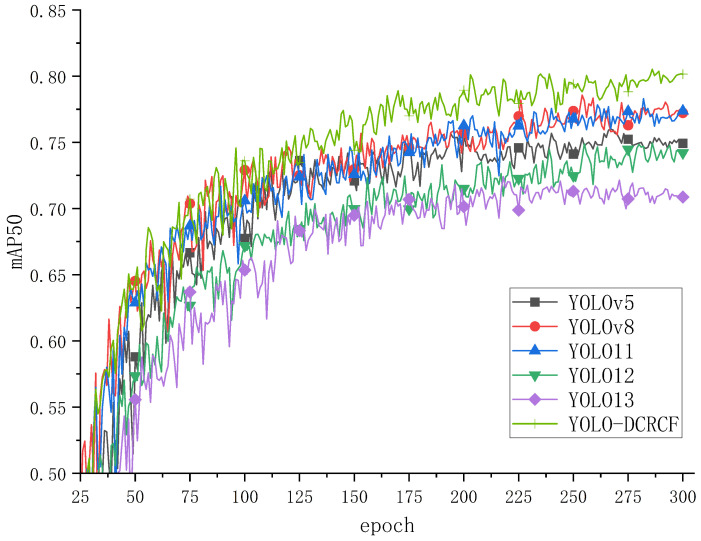
The model’s training on the SHAGWD yielded the shown mAP50 values.

To evaluate the efficacy of the YOLO-DCRCF model, proposed in the present study, in detecting safety helmet and glove wearing within power grid operation environments, comparative experiments were performed on the SHAGWD, with the training mAP50 curves for various models depicted in Figure 7.

Figure 7 depicts the training comparison curves for various models on the SHAGWD, where the horizontal axis represents the iteration counts and the vertical axis denotes mAP50 validation at 25-epoch intervals. The analysis is as follows. The YOLO-DCRCF model, proposed in the present study, significantly surpassed the other baseline models in terms of mAP50, thus confirming the efficacy of the proposed model for safety helmet and glove wearing detection in power grid operation environments.

Table 3 depicts the performance test results for various models on the SHAGWD for safety helmet and glove wearing detection in power grid operation environments, with the analysis being as follows. The YOLO-DCRCF model, proposed in the present study, demonstrated enhancements of 3.9% in precision, 2.0% in recall, 2.7% in mAP50, and 0.8% in mAP50-95 relative to the baseline YOLO11 model. Although the YOLO11 model showed a marginal decline in recall and mAP50 relative to YOLOv8, the YOLO-DCRCF model achieved enhancements of 1.3% in recall and 2.4% in mAP50 compared with YOLOv8.

### 5.4. Ablation Experiment

In order to verify the contribution of the DCNv2 module and the RCF module to the model’s performance, detailed ablation experiments were designed on the SHWD and the SHAGWD in this paper.

**Figure 8:**
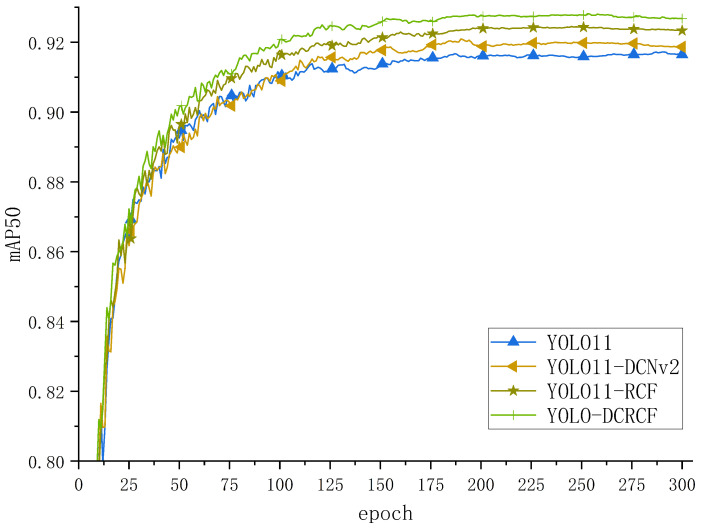
The comparison curves of ablation experiments between the model proposed in this paper and the baseline model on the SHWD.

Figure 8 depicts the training curves for various models on the public Safety Helmet Wearing Dataset (SHWD), with the analysis being as follows. Relative to the baseline YOLO11 model, YOLO11-DCNv2 and YOLO11-RCF exhibited enhanced mAP50 performance, confirming that the integration of DCNv2 improved YOLO11’s geometric transformation modeling, thereby enhancing its safety helmet detection capabilities. Moreover, following integration of the newly proposed RCF network, the model dynamically adjusts feature weights based on the diverse resolutions and contents of feature maps, precisely capturing the target’s multi-scale features, thus improving overall model performance.

Table 4 displays the test results of the model on the public SHWD, and the detailed analysis is as follows:

1. Relative to the baseline YOLO11 model, the YOLO11-DCNv2 model exhibited a 0.5% reduction in recall while demonstrating enhancements of 0.5% in precision, 0.4% in mAP50, and 1.1% in mAP50-95. These findings confirm that integrating convolutional layers with offset learning capabilities into the YOLO11 network facilitates sampling control across a wider range of feature levels; moreover, the incorporation of a modulation mechanism further strengthens the network’s capacity to manipulate spatial support regions.
2. Relative to the baseline YOLO11 model, the YOLO11-RCF model exhibited a 0.1% reduction in recall while demonstrating enhancements of 0.7% in precision, 0.4% in mAP50, and 0.7% in mAP50-95. These findings confirm that the YOLO11-RCF model, through the integration of the Selective Boundary Aggregation (SBA) module, effectively aggregates boundary and semantic information, thereby enhancing multi-scale feature fusion and improving overall model performance.

**Figure 9:**
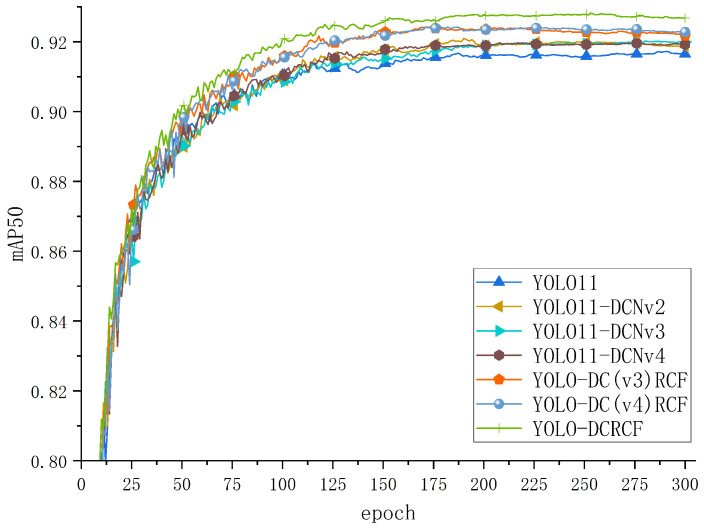
Training curves of model integration with various versions of deformable convolution.

To evaluate the efficacy of the proposed second version of deformable convolution, the present study compared it against the third and fourth versions of deformable convolution, with the experimental results depicted in Figure 9.

Figure 9 indicates that the training performance of the second version of deformable convolution surpassed that of the third and fourth versions. The analysis is as follows:

1. Relative to the baseline YOLO11 model, the networks integrating deformable convolution exhibited higher mAP50 values, with comparable convergence curves across all three versions, confirming that the integration of deformable convolution into YOLO11 improved model performance.
2. The integration of the RCF structure into the three versions of deformable convolution networks further enhanced mAP50 performance, with the YOLO-DCRCF model surpassing the YOLO-DC(v3)RCF and YOLO-DC(v4)RCF models in the convergence curves, confirming that the second version of deformable convolution was more compatible with the RCF network.

Table 5 presents the test results of YOLO11 integrated with different versions of deformable convolutional networks. A detailed analysis is provided below. Compared with YOLO-DC(v4)RCF, YOLO-DCRCF exhibited a decrease of 0.8% in precision and 0.2% in mAP50-95, primarily due to an increase in false positives and slightly reduced localization accuracy. Nevertheless, YOLO-DCRCF demonstrated improvements of 1% in recall and 0.3% in mAP50 over YOLO-DC(v4)RCF, indicating superior capability in detecting all targets within the image and delivering higher average precision at lower intersection over union (IoU) thresholds. For power grid operation environments such as security surveillance, where missed detections are critical, YOLO-DCRCF proves to be a more advantageous option. In contrast, for tasks with low tolerance for false positives or requiring high-precision bounding box localization (e.g., industrial quality inspection or drone-based aerial analysis), YOLO-DC(v4)RCF is more suitable.

**Figure 10:**
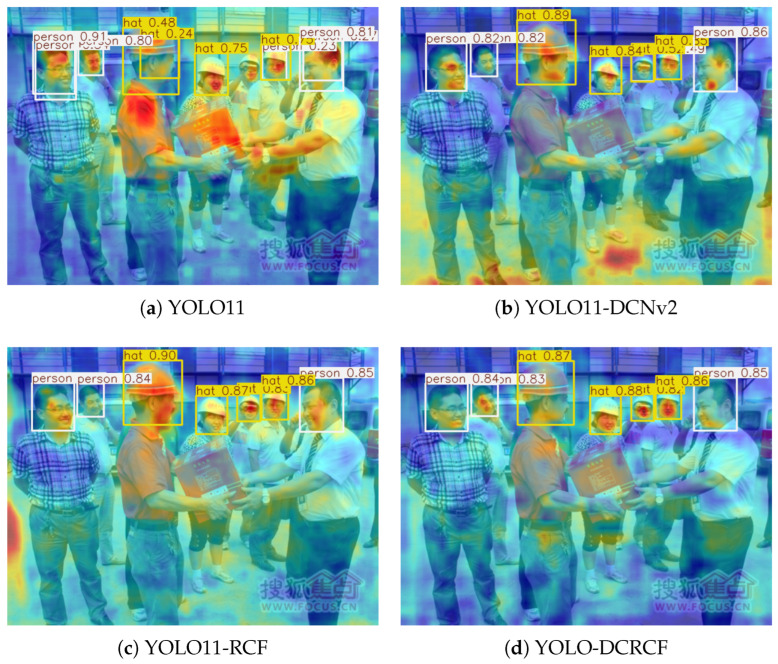
Comparison of heatmap results of different models (**a**–**d**) during the testing process.

Figure 10 illustrates the heatmap comparison results of various models on the public SHWD, based on the optimal weights obtained during training. The comparative analysis indicates that, compared with the baseline model YOLO11, the other three models successfully detected all persons and safety helmets. Despite retaining object detection capabilities, the YOLO11-DCNv2 model exhibited abnormally dispersed heatmap responses, with insufficient feature focus in critical regions such as helmets. The YOLO11-RCF model demonstrated enhanced feature extraction capability, and its heatmap distribution was relatively concentrated, though some activation still spread into non-target areas. A comprehensive comparison shows that the YOLO11-DCRCF model exhibited a distinct advantage in heatmap visualization; its feature activations were highly focused on the target head region (safety helmets), with the least amount of noise responses spreading to irrelevant regions, suggesting optimal performance in both object localization accuracy and feature representation capacity.

**Figure 11:**
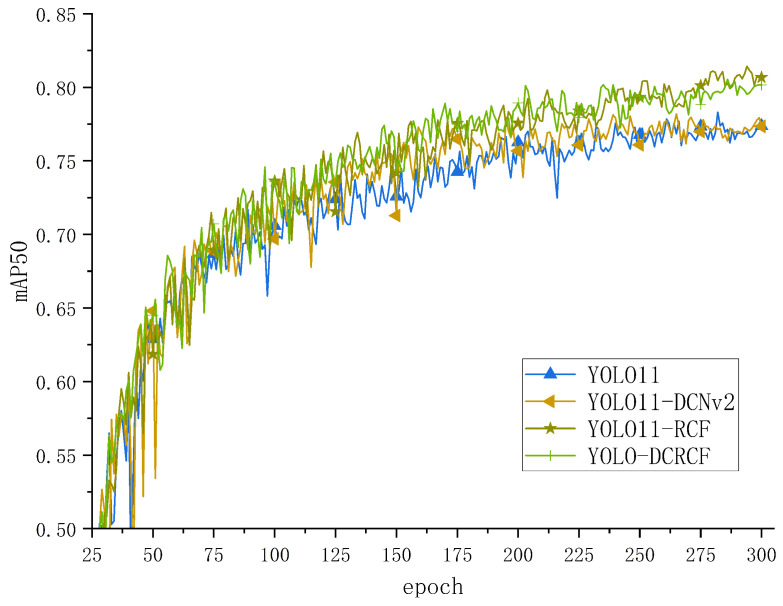
The comparison curves of ablation experiments between the model proposed in this paper and the baseline model on the SHAGWD.

Figure 11 depicts the training curves of the model on the SHAGWD for the detection of safety helmet and glove wearing in the electrical grid work environment. The specific analysis is as follows. Compared with the baseline model, YOLO11-DCNv2 and YOLO11-RCF achieved further improvements in mAP50, demonstrating that the modules proposed in this paper can effectively enhance the model’s detection capability for the wearing of safety helmets and gloves in the electrical grid work environment, thus validating the effectiveness of the model.

Table 6 presents the test results of the model on the SHAGWD for the detection of safety helmet and glove wearing in the electrical grid work environment. The specific analysis is as follows:

1. For DCNv2, compared with the baseline model, YOLO11-DCNv2 achieved improvements of 1.4% in precision, 0.7% in recall, and 1.1% in mAP50. However, there was a decrease of 0.2% in mAP50-95, which can be attributed to overfitting to certain features during the training process caused by the introduction of deformable convolutions in DCNv2.
2. For RCF, compared with the baseline model, YOLO11-RCF yielded improvements of 1.5% in precision, 1.8% in recall, and 1.4% in mAP50. Nevertheless, there was a decrease of 0.3% in mAP50-95, which was due to the prediction bounding box offsets at high IoU thresholds leading to a decline in mAP50-95.
3. By integrating DCNv2 and RCF, compared with the baseline model, YOLO-DCRCF showed improvements across all metrics, demonstrating that the two modules proposed in this paper are suitable for YOLO11, reflecting the excellent robustness and effectiveness of YOLO-DCRCF.

## 6. Conclusions

To improve the detection performance of safety helmet and glove wearing in complex power grid operation environments, this paper proposed YOLO-DCRCF, a detection algorithm specifically designed for such scenarios. Specifically, to address the detection challenges posed by the irregular shapes of safety helmets and gloves, a deformable convolutional network (DCN) was employed to enhance the network’s capability in modeling geometric transformations. Comparative experiments with three different DCN variants revealed that DCNv2 was most compatible with the proposed algorithm. In addition, to better localize the boundaries of safety helmets and gloves, a recalibrated feature pyramid (RCF) network was designed. By introducing a bidirectional fusion mechanism between high- and low-resolution features, the network facilitated more effective information exchange across scales and improves multi-scale feature integration. Moreover, an adaptive attention mechanism was incorporated to dynamically adjust feature weights according to the resolution and content of feature maps, thereby capturing multi-scale object features more effectively. The experimental results demonstrate that the proposed YOLO-DCRCF achieved superior performance compared with baseline models on both the Safety Helmet Wearing Dataset (SHWD) and the Safety Helmet and Gloves Wearing Dataset (SHAGWD) collected in power grid operation environments, indicating its suitability for detecting safety helmet and glove wearing in complex power grid scenarios.

## Figures and Tables

**Table 1:** Experimental environment.

Experimental Environment	Environment Configuration
Operating systems	Win11
CPU	Ryzen 9 7950X
Video Cards	GeForce RTX 4090
RAM	64 GB
Storage	2 TB SSD
Programming Languages	Python 3.10
Framework	Pytorch 2.0.1

**Table 2:** The measurement results of each model’s speed on the Safety Helmet Wearing Dataset (SHWD) after 300 epochs. Bold values indicate the best performance in each column.

Model	Precision	Recall	mAP50	mAP50-95	Inference (ms)	FLOPs (G)
YOLOv5	0.906	0.870	0.913	0.583	2.0	5.8
YOLOv8	0.897	0.866	0.910	0.578	1.7	6.8
YOLO11	0.902	0.876	0.916 (±0.0115)	0.585 (±0.0106)	1.7	6.3
YOLO12	0.907	0.861	0.914	0.591	2.6	6.1
YOLO13	0.916	0.851	0.910	0.586	2.9	6.1
YOLO-DCRCF	**0.909**	**0.885**	**0.927** (±0.0133)	**0.606** (±0.0.0107)	2.9	13.4

**Table 3:** The test results of each model after training for 300 epochs on the Safety Helmet and Gloves Wearing Dataset (SHAGWD). Bold values indicate the best performance in each column.

Model	Precision	Recall	mAP50	mAP50-95
YOLOv5	0.836	0.701	0.757	0.442
YOLOv8	0.840	0.730	0.772	0.461
YOLO11	0.851	0.723	0.769	0.464
YOLO12	0.787	0.734	0.748	0.437
YOLO13	0.771	0.683	0.715	0.425
YOLO-DCRCF	**0.890**	**0.743**	**0.796**	**0.472**

**Table 4:** The test results of the model proposed in this paper and the baseline model after 300 epochs on the Safety Helmet Wearing Dataset (SHWD). Bold values indicate the best performance in each column.

Model	Precision	Recall	mAP50	mAP50-95	Inference (ms)	FLOPs (G)
YOLO11	0.902	0.876	0.916	0.585	1.7	6.3
YOLO11-DCNv2	0.907	0.871	0.920	0.596	2.3	6.3
YOLO11-RCF	0.909	0.875	0.920	0.592	2.8	13.5
YOLO-DCRCF	**0.909**	**0.885**	**0.927**	**0.606**	2.9	13.4

**Table 5:** Test results for model integration with various versions of deformable convolution. Bold values indicate the best performance in each column.

Model	Precision	Recall	mAP50	mAP50-95
YOLO11	0.902	0.876	0.916	0.585
YOLO11-DCNv2	0.907	0.871	0.920	0.596
YOLO11-DCNv3	0.910	0.871	0.914	0.585
YOLO11-DCNv4	0.910	0.871	0.919	0.598
YOLO-DC(v3)RCF	0.910	0.877	0.920	0.597
YOLO-DC(v4)RCF	0.910	0.871	0.919	0.598
YOLO11-RCF	**0.917**	0.875	0.924	**0.608**
YOLO-DCRCF	0.909	**0.885**	**0.927**	0.606

**Table 6:** The test results of the model proposed in this paper and the baseline model after 300 epochs on the SHAGWD. Bold values indicate the best performance in each column.

Model	Precision	Recall	mAP50	mAP50-95
YOLO11	0.851	0.723	0.769	0.464
YOLO11-DCNv2	0.865	0.730	0.780	0.462
YOLO11-RCF	0.866	0.741	0.783	0.461
YOLO-DCRCF	**0.890**	**0.743**	**0.796**	**0.472**

## Data Availability

The original contributions presented in this study are included in the article. Further inquiries can be directed to the corresponding author.
